# A Web-Based Platform for Patients With Osteoarthritis of the Hip and Knee: A Pilot Study

**DOI:** 10.2196/resprot.5665

**Published:** 2016-06-03

**Authors:** Leif E Dahlberg, Daniel Grahn, Jakob E Dahlberg, Carina A Thorstensson

**Affiliations:** ^1^ Orthopedics Department of Clinical Sciences Lund, Skåne University Hospital Lund University Lund Sweden; ^2^ Joint Academy Malmö Sweden; ^3^ Institute of Neuroscience and Physiology, Sahlgrenska Academy Department of Clinical Neuroscience and Rehabilitation University of Gothenburg Gothenburg Sweden; ^4^ BOA Registry Registercentrum Västra Götaland Gothenburg Sweden

**Keywords:** osteoarthritis, exercise therapy, mobile apps, digital therapeutics

## Abstract

**Background:**

Chronic conditions are the leading cause of disability throughout the world and the most expensive problem facing the health care systems. One such chronic condition is osteoarthritis (OA), a frequent cause of major disability.

**Objective:**

To describe the effect on joint pain for the first users of a newly developed Web-based osteoarthritis self-managing program, Joint Academy, and to examine whether these patients would recommend other OA patients to use the program.

**Methods:**

Patients with clinically established knee or hip OA according to national and international guidelines were recruited from an online advertisement. A trained physiotherapist screened the eligible patients by scrutinizing their answers to a standardized questionnaire. The 6-week program consisted of eight 2- to 5-minute videos with lectures about OA, effects of physical activity, self-management, and coping strategies. In addition, exercises to improve lower extremity physical function were introduced in daily video activities. During the course of the program, communication between physiotherapist and patients was based on an asynchronous chat. After 6 weeks, patients were able to continue without support from the physiotherapist. Patients reported their current pain weekly by using a numeric rating scale (range 0-10; 0=no pain, 10=worst possible pain) as long as they were in the program. In addition, after 6 weeks patients answered the question “What is the probability that you would recommend Joint Academy to a friend?”

**Results:**

The eligible cohort consisted of 53 individuals (39 women; body mass index: mean 27, SD 5; age: mean 57, SD 14 years). With the continued use of the program, patients reported a constant change in pain score from mean 5.1 (SD 2.1) at baseline to mean 3.6 (SD 2.0) at week 12. Six patients participated for 30 weeks (mean 3.2, SD 2.1). Overall, the patients would highly recommend Joint Academy to other OA patients, suggesting that the platform may be useful for at least some in the vast OA population.

**Conclusions:**

Joint Academy, a Web-based platform for OA therapy, has the potential to successfully deliver individualized online treatment to many patients with OA that presently lack access to treatment.

## Introduction

Chronic conditions are the leading cause of disability throughout the world and collectively they represent the most expensive problem facing health care systems [1]. One prevalent condition among these noncommunicable diseases is osteoarthritis (OA), which is one of the leading causes of global disability [2]. Approximately 27 million individuals live with OA in the United States [3], estimated to cost US $189 billion annually [4,5] highlighting the financial and societal burden attributed to OA.

Osteoarthritis primarily affects the elderly. The prevalence in the age group 60 years and older is 10% among men and 18% among women [6]. Ongoing demographic changes, particularly in developed countries, and a growing number of elderly individuals suggest that the number of people with OA will increase.

Like other chronic diseases, OA progresses slowly. Before individuals are eligible for total joint replacement (TJR) surgery, incubation time is some 10 to 15 years with increasing joint pain, decreasing function, and reduced quality of life. Accordingly, cross-sectional Swedish and UK data show that only 20% of the OA population qualifies for TJR in spite of debilitating symptoms [7-9]. For those in earlier stages of OA when the diagnosis should be based on clinical symptoms, the primary treatment is nonsurgical, based on exercise, information, and—in relevant cases—weight loss according to all international and national guidelines [10-13]. Unfortunately, this evidence-based treatment is not reflected in present OA management administered by the health care system [14]. Rather, joint pain is considered by health care professionals as a normal part of the aging process (ie, “wear and tear of the body”) and is therefore not manageable until TJR becomes an option. As a result, many suffering from OA are not aware of or offered well-established evidence-based nonsurgical treatment.

The Swedish national initiative “Better Management of Patients with OsteoArthritis” (BOA), a national quality registry and an evidence-based supported self-management program for patients with OA, was developed to facilitate the implementation of guidelines [9,14]. Of Sweden’s 9 million inhabitants, more than 50,000 individuals have participated in the BOA program between 2008 and 2015. Still, they represent less than 20% of people in need of treatment due to joint problems [9,14]. Thus, despite the systematic and thorough work put into BOA, most individuals suffering from OA have not yet received access to the program, which may be due to lack of health care resources or people having trouble fitting their schedule to primary care opening hours. Therefore, alternative methods are required to reach these individuals.

One appealing method that leverages technology into health care is digital therapeutics. In this context, digital therapeutics can be viewed as software functioning as “medication” that is delivered via the Web. This treatment focuses on behavioral changes with long-term improvements in contrast to the short-term gain of taking a pill or other interventions presently used in health care. A crucial point to achieve the effect of any treatment is compliance. Using digital therapeutics that utilize the Internet to deliver cost-effective treatment around the clock has the potential to increase adherence. Allowing for people to administer their treatment at a suitable time point probably increases compliance and the likelihood of improved health and quality of life for patients with chronic conditions. An interesting and successful example is the translation of the Diabetes Prevention Program into an online treatment [15,16]. With respect to OA, we have developed Joint Academy [17], which is a digital platform for individuals with clinically verified OA. The platform is based on the BOA program [9,11,14]. It includes a Web-based patient interface that provides individualized exercises, a personal physiotherapist, peer-to-peer support, education about lifestyle and behavioral changes, and a physiotherapist interface that provides necessary information on the patient’s progress in the program for support and encouragement.

The aim of this pilot study was to describe the effect on joint pain for the first users of a newly developed Web-based OA self-managing program, Joint Academy, and to examine whether these patients would recommend other OA patients to use the program. Collectively, the objectives aimed at deciding (1) whether it seemed feasible to deliver Web-based OA treatment and (2) whether the results would support further development of the platform.

## Methods

### Patients and Study Design

Participants with knee or hip joint pain were recruited from an online advertisement on the home page of the Swedish Rheumatology Association during two weeks in January 2015. However, single patients were eligible for inclusion until December 2015. The potential participants were directed to a website where they were asked to create a user account and fill in a screening questionnaire. Participants were not compensated for their participation but were enrolled in the program at no cost.

A trained physiotherapist (CT) screened the questionnaires and, whenever relevant, asked additional questions to patients through their user interface. To be included, the physiotherapist ensured that described symptoms were in agreement with clinical OA according to national guidelines [9,11,14]. Exclusion criteria included chronic widespread pain or other, more severe diseases, such as inflammatory joint disease, cancer, sequel after hip fracture, or due to major trauma.

Participants were informed that the program lasts for 6 weeks. Included participants (referred to as patients subsequently) were asked to answer some demographic questions according to the International Consortium for Health Care Measurement (ICHOM) initiative as well as those in the BOA registry [9,14,18] and reported their current baseline of pain using the Numeric Rating Scale (NRS; range 0-10, 0=no pain, 10=worst possible pain). After 6 weeks, patients were asked “What is the probability that you would recommend Joint Academy to a friend?” (range 0-10, 0=not likely, 10=most likely). For this study, patients were not asked to specify what joint was affected by their disease.

### Description of the Intervention

The basis for Joint Academy is the Supported Osteoarthritis Self-management Program (SOASP) used in BOA. The BOA program consists of theory sessions held by a physiotherapist, sometimes in collaboration with an occupational therapist and an OA communicator (ie, a patient with OA who has been educated by the Swedish Rheumatology Association to talk about the daily experience of OA and good coping strategies including physical activity). After completing the theory sessions, patients can opt for an individually adapted and physiotherapist-supervised exercise program [14]. The SOASP content was based on existing evidence, national and international treatment guidelines, as well as patients’ views, thoughts, and tolerability of treatment and exercise for OA. Patients in the SOASP rate their pain on a visual analog scale at baseline and again after 3 and 12 months.

The Joint Academy program that was used in this study started on Sunday and ran for 6 weeks. The program consisted of eight videos of 2- to 5-minute lectures about OA, effects of physical activity, self-management, and coping strategies. After each lecture, the patient took a quiz to confirm that the take-home messages of the lecture were correctly understood. Parallel to these lectures, four neuromuscular exercises were introduced to improve lower extremity physical function. Each exercise had 3 to 5 levels of intensity. The level of intensity was based on an algorithm taking into account individual progress and the patient’s perceived ability to perform the exercise without exacerbating pain. The week’s exercises and lessons (12-14 exercises, two lessons, and one pain report per week) were divided into daily packages and delivered in video format to the patient during the 6-week period by push email. In each email, there was a link to the embedded videos within the Web-based platform. These videos showed how to properly perform the exercises. The short video lectures also included key OA issues important for understanding the delivered treatment to be fully motivated for the exercises. Each package was designed to take no more than 5 to 15 minutes per day. After having performed an exercise, the patient registered it as complete. When needed, the patient was able to communicate questions to the personal physiotherapist. This communication between the physiotherapist and patient within the Joint Academy platform was asynchronous and based on a chat during the 6-week program. To have a comparable benchmark over time, pain was always reported on Sundays and referred to the average pain during the week. An “active week” was defined as a week when patients reported their pain level. If a patient reported pain values for four consecutive weeks, skipped two weeks, and finally reported pain for three additional weeks, this was defined as seven active weeks in the program.

### Software Programming

The software was compatible with all platforms and worked on personal computers, tablets, and mobile phones. It was built as a single-page Web app with a responsive user interface to facilitate user experience. The Web app was connected to our proprietary back-end service for OA treatment. The back-end was built on the framework Ruby on Rails and the front-end on Angular JS.

### Statistical Analyses

The statistical analysis was performed using a longitudinal random effects model. A random intercepts and slopes model was fitted with using the restricted maximum likelihood approach and with the underlying assumption of an unstructured variance-covariance matrix and degrees of freedom estimated using Satterthwaite’s method. The calculations were performed using the mixed command in Stata version 14.

### Ethical Consideration

Patients gave informed consent when entering the program.

## Results

The study cohort consisted of 53 individuals (39 women; body mass index [BMI] mean 27, SD 5; age mean 57, SD 14 years). Of these 53 patients, 36 (68%) registered their pain levels for 6 active weeks (Table 1). On average, patients needed 7 to 8 weeks to complete a 6-week active period (Table 1). During these weeks, patients received 113 activities in total, of which they performed a mean 83 (SD 13) activities.

**Table 1:** Study results summary.

Result	Number of active weeks					
	Baseline	6	12	18	24	30
Number of patients in program, n	53	36	19	12	9	6
Time to complete active weeks (days), mean (SD)		53 (18)	112 (53)	154 (32)	201 (39)	246 (49)
Activities per week, mean (SD)		14 (2)	13 (2)	13 (2)	14 (1)	14 (1)
NRS pain score, mean (SD)^a^	5.1 (2.1)	4.5 (1.8)	3.6 (2.0)	3.3 (2.5)	2.7 (1.7)	3.2 (2.1)
Change in mean NRS pain score vs baseline, %		–11	–28	–35	–47	–38
Patients with >15% improvement in NRS pain score, n (% of remaining patients) [19]		17 (47)	12 (63)	7 (58)	8 (89)	5 (83)

^a^ Numeric Rating Scale: range 0-10.

We observed that 33 patients voluntarily continued using the program after 6 weeks utilizing the same weekly instructions as in week 6, but without the support of a personal physiotherapist.

**Figure 1:**
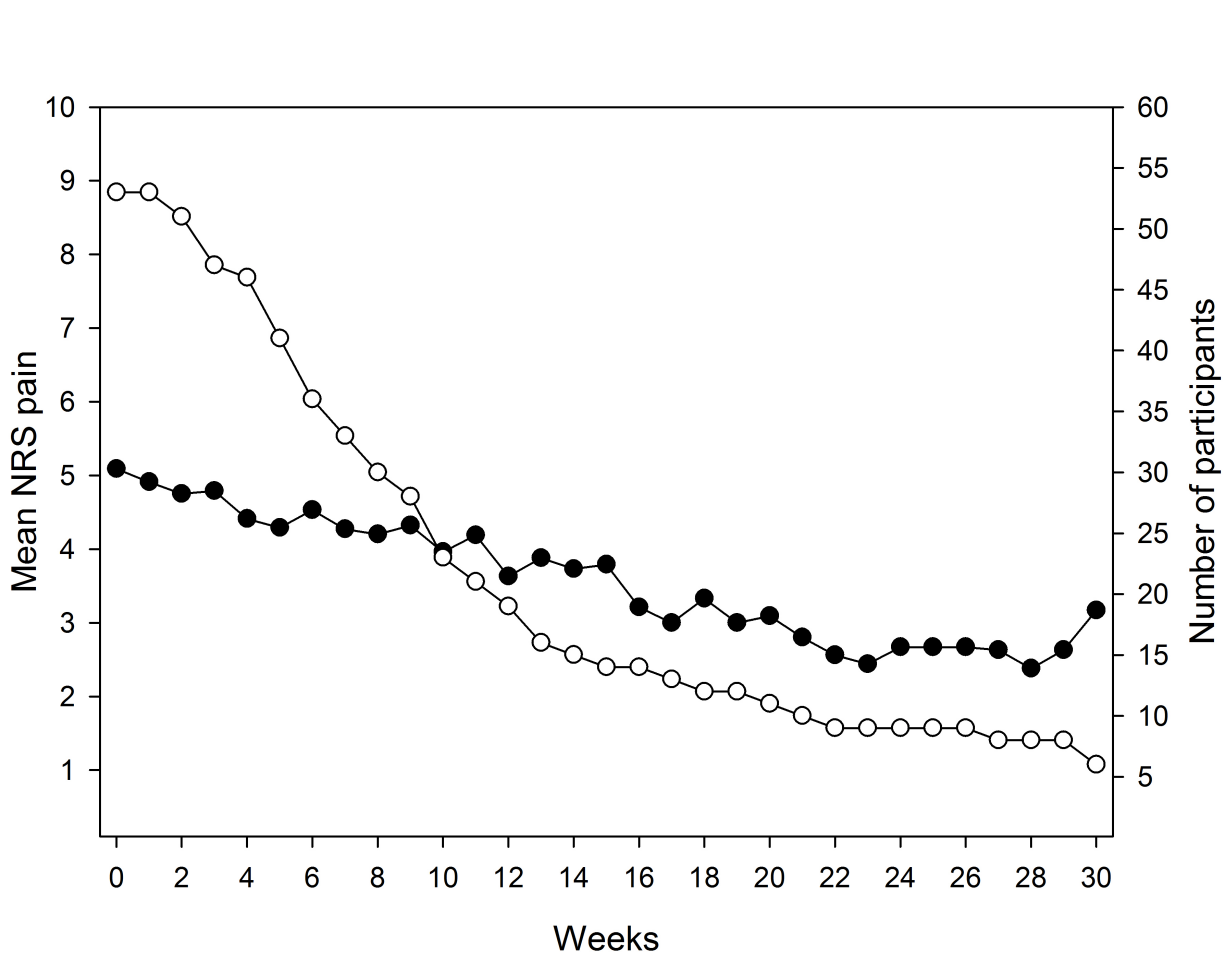
Mean NRS pain score for active patients (●) and number of patients (○) remaining in the program at each week. Due to decreasing number of patients during the course of the program, individual weekly changes may have a disproportional effect on the mean pain level.

**Figure 2:**
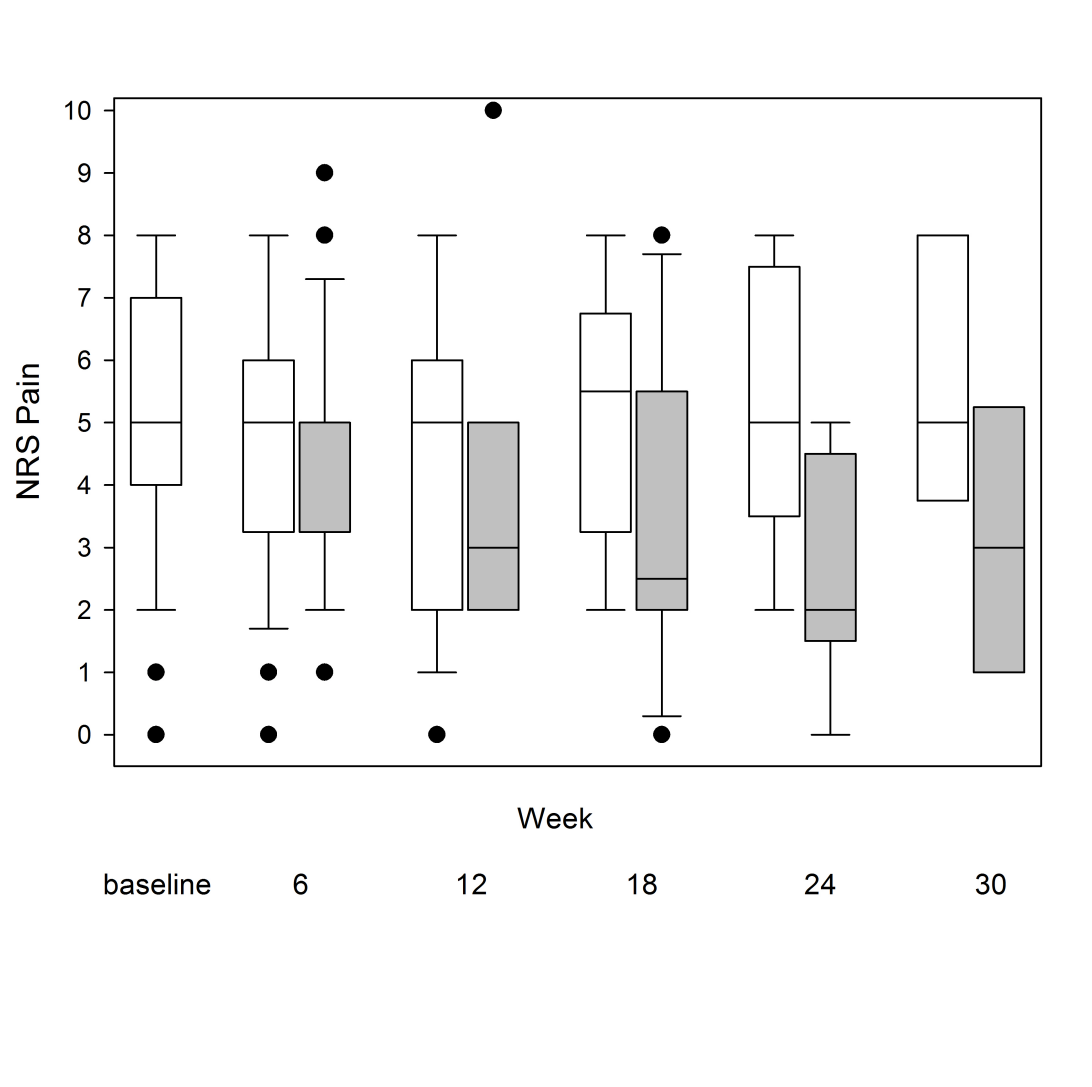
Box plots of the NRS pain values (the horizontal line in the middle of each box indicates the median and the top and bottom borders of the box mark the 75^th^ and 25^th^ percentiles, respectively; the whiskers above and below the box mark the 90^th^ and 10^th^ percentiles; the black dots beyond the whiskers are outliers) at different time points. Baseline is baseline mean NRS for all 53 patients. For each subsequent time point, data are presented for those patients that participated in the program at the indicated time period (6 weeks: n=36; 12 weeks: n=19; 18 weeks: n=12; 24 weeks: n=9; 30 weeks: n=6). White boxes: baseline NRS; gray boxes: NRS after the indicated time period.

**Figure 3:**
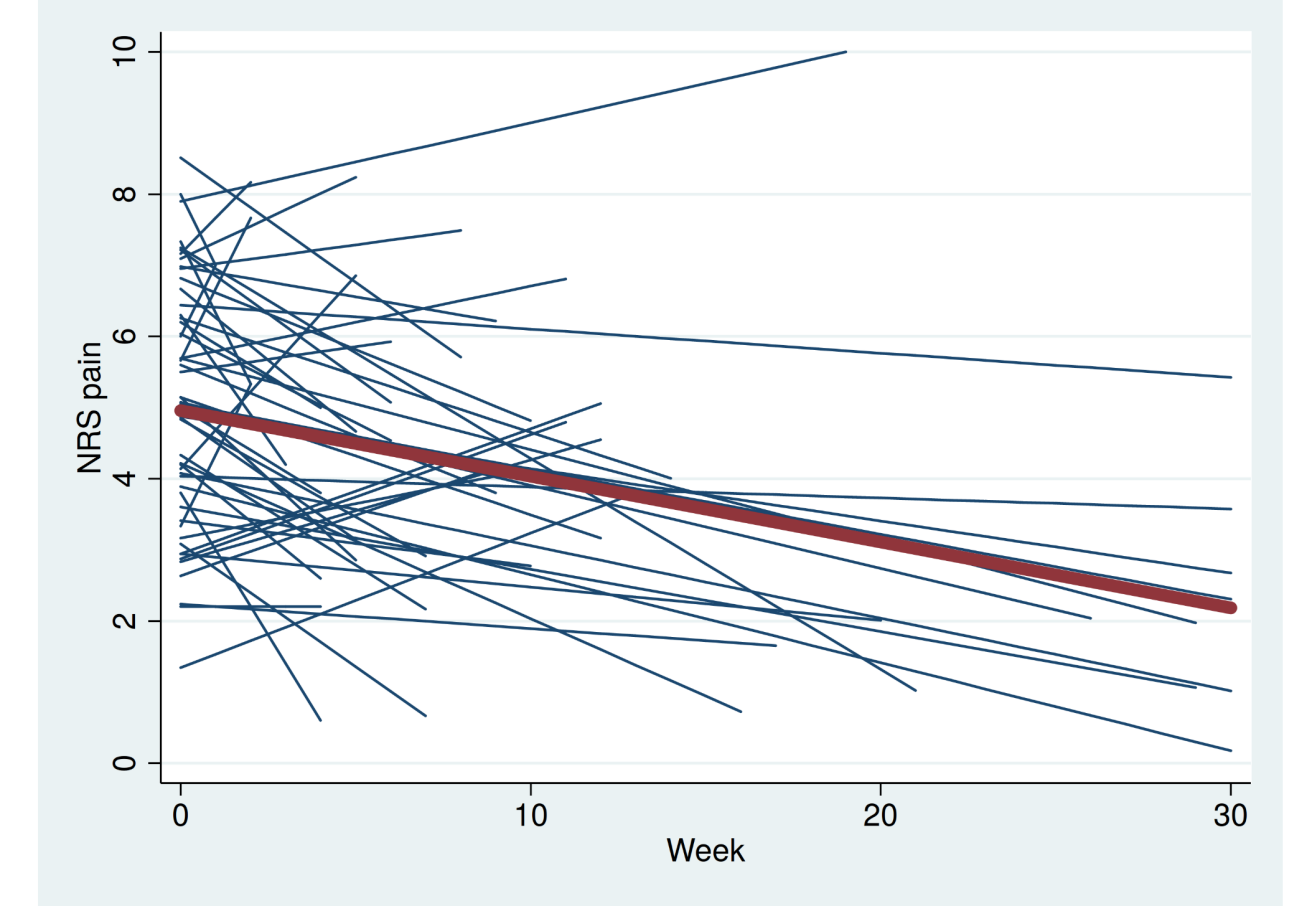
Spaghetti plot showing NRS pain. Each blue line represents a single patient. The red line represents change in mean pain over time for all 53 patients.

Furthermore, 19 patients (36%) voluntarily continued to exercise and report their pain level for a total of 12 active weeks. After 18 and 24 active weeks, there were 12 and nine patients remaining, respectively, and six patients completed 30 active weeks in the program (Figures 1 and 2,Table 1). The longest participating patient continued for a total period of 50 weeks (data not shown). Pain according to the NRS was markedly reduced over a 30-week period (Figures 1-3,Table 1). The mean weekly change in pain during follow-up was estimated to be –0.074 (95% CI –0.118 to –0.030, *P*=.002), which corresponds to a pain reduction of one unit every 14 weeks.

Figure 2 shows that baseline values were similar regardless of how long patients participated in the program (ie, patients engaged in the program for a longer time period did not have less severe pain at baseline compared to baseline values for patients who participated for a shorter period of time). There were no obvious demographic differences (ie, sex, age, BMI) between patients who were active for less than 6 weeks compared to those who were active for more than 6 weeks (data not shown). At all weeks during the 30-week period, the mean pain score of the active patients was similar to the reported pain score of the patients that discontinued the program after that week (data not shown).

At week 6, 18 of the 36 active patients had a lower absolute value in pain score, eight were unchanged, and 10 had an increase compared to baseline. The mean NRS pain score for the 36 active patients changed from mean 5.1 (SD 2.1) at baseline to mean 4.5 (SD 1.8) (Table 1). Sixteen patients (68%) were classified as responders with an individual improvement of more than 1.5 in NRS pain score [19].

The NRS pain data showed that at weeks 6 and 30, 47% (17/36) and 83% (5/6) were responders, respectively (Table 1).

In all, 31 patients (five were lost due to technical reasons) answered the question “What is the likelihood that you would recommend Joint Academy to a friend?” The median score was 10 (range 6-10).

## Discussion

In this study, we demonstrate that Joint Academy, a Web-based platform for OA therapy, has the potential to successfully deliver individualized digital treatment to patients with clinical OA in the hip or knee. Many experienced an improvement in pain and would recommend the program to others. This is one of few exercise intervention studies following patients for a longer period of time [20,21]. This study shows similar pain improvement as those in previous face-to-face studies [13,14,22,23]. Furthermore, although a definitive cause relationship could not be established, the degree of improvement seems to be associated with duration of stay in the program. Although not designed to determine reasons for discontinuing the program, the study revealed that patients who discontinued the program within 6 weeks showed similar pain scores at baseline (Figure 2) compared to those that remained in the program. Of note, patients consecutively entered the program during the 12 months of recruitment meaning that not all patients had the opportunity to participate for 30 weeks and that the lower number of patients by time is not a true measure of compliance.

Even though several patients discontinued the program, results suggest that pain fluctuates over time (Figure 1). This is in agreement with the clinical profile of OA showing relapsing intervals of pain and impaired function. Another possible explanation for the fluctuating pain level may be that as patients improve (ie, their symptoms become less severe), they increase exercise time and intensity. This suggests that patients may have similar or increased pain but, at the same time, they have increased physical function. Future studies will explore the relationship between pain and function by assessing physical function, which was recently included in Joint Academy.

In SOASP, patients rate their pain on a visual analog scale (VAS) at baseline and again after 3 and 12 months [13,14]. On average, the VAS pain score decreases by 10 points (from 48 to 38) and 12 points (from 48 to 36) for patients with hip and knee OA, respectively. These results are similar to the results reported at 12 weeks in this study (5.1 to 3.6) (Table 1) indicating that a Web-based means to deliver evidence-based health care to OA patients seems to work as well as the “analog” face-to-face predecessor.

In the United States, US $40 billion per year is allocated to the more than 600,000 TJR operations conducted annually, making TJR one of the most expensive interventions today [4]. The number of TJRs is expected to increase by more than 100% by 2030 due to the increasing prevalence of OA in an aging population, together with the decreasing age of intervention for TJR in the baby boomer generation [24]. Without doubt, TJR is a very successful intervention when performed on the right patient at the right time point. However, recent studies have shown that many TJRs, as well as other surgical interventions in patients with OA, are often unnecessary and that indication for surgery is not well validated. For instance, a study that compared pre- and post-health care costs for OA patients that underwent a TJR in the United States showed that although the total number of outpatient visits declined after surgery, the percentage of patients hospitalized after TJR increased. The result was a higher total cost during follow-up compared to before surgery [25]. In addition, TJR patients may need revision surgery (ie, a new prosthesis) and TJR is associated with an increased risk for adverse events compared to nonsurgical treatment [24]. Furthermore, 15% to 20% of the TJR population has sustained disabilities after surgery, which generates suffering, costly visits, and unnecessary diagnostics and treatments [26].

Another common operative procedure in middle-aged patients with knee pain is arthroscopy. In the United States alone, 400,000 arthroscopies are performed annually due to the popular belief that pain in the degenerative knee is caused by a meniscal tear [27-30]. As concluded by Katz and Jones [28], a reasonable initial strategy for these patients is physiotherapy rather than arthroscopy. This conclusion is supported by a recent study that contradicts the prevailing consensus that mechanical symptoms justify an arthroscopic intervention [31]. Furthermore, partial meniscectomy may be associated with increased risk of incident radiographic osteoarthritis [32]. That physiotherapy indeed has an effect, and that the results of this study shows similar effect, is further demonstrated by a recent Cochrane review [22] as well as results from the Danish GLA:D program, which is based on the BOA program [23]. The positive effect of exercise, weight control, and information can be explained by the biomechanical origin of OA as well as the importance of patients having accurate knowledge about their disease [22,33].

Two randomized controlled trials are of interest with respect to nonsurgical options to treat OA patients eligible for total knee replacement [34,35]. One of these studies showed that supervised exercise before surgery is associated with a faster postoperative recovery [34]. The second study compared knee TJR with a nonsurgical treatment program and showed substantial improvement in both groups with respect to most outcomes. However, only 26% of the patients who were assigned to receive nonsurgical treatment alone underwent total knee replacement in the year following the procedure [35]. That education and individually adapted exercise have the potential to reduce the need for TJR is further enforced by Svege et al [36].

To our knowledge, Joint Academy is the first platform to deliver digital health care to OA patients. The fact that the program may reverse the course of symptoms for some patients to the degree observed in the current study is very encouraging, suggesting that Joint Academy may be feasible at least for some OA patients. For the subgroup of the OA population volunteering for participation in this program, a pragmatic approach with 5 to 15 minutes of exercise a few days per week seemed sufficient to achieve significant results. Interestingly, the program motivated the patients to perform approximately 80 activities in a 7- to 8-week period. This suggests that Joint Academy may play an important role in OA treatment. In addition, Joint Academy may also increase equity in OA treatment by offering evidence-based health care for people living with clinical hip or knee OA in the developing world.

That a digital health program may have significant effects on health is also shown by the Prevent program targeting patients with prediabetes. By combining weekly theoretical lessons and individualized health coaching, patients lowered their body weight as well as their blood glucose levels [15,16]. The great advantage of a Web-based platform that works on personal computers, tablets, and mobile phones, is that it can be used wherever and at a time point of the patient’s own choice, minimizing the interruption of daily life activities and the need for scheduled appointments at a clinic. This may be particularly relevant for patients in rural areas with limited access to and/or living far away from health care facilities as well as for working people who may find it difficult to allocate time for a visit to a primary care practice. In addition, Internet availability is increasing rapidly as the price of a basic mobile phone decreases. Ultimately, digital health care may save financial resources and increase quality of life for many people living with chronic diseases.

There are limitations to this study. This is a pilot study without a control group and with a small study population, especially at later time points, limiting the establishment of a definitive cause relationship between length of participation in Joint Academy and improvement in pain. However, patient attrition over time is not due to demographic differences between those patients who discontinued the program and those patients that continued the program. When using this study design, there is a risk that the cohort is not representative of the general OA population. However, both the OA pathology and the clinical disease patterns are similar around the world. The relative ease with which you can enter the study (eg, patients do not need to visit a general physician to have a diagnosis or a physiotherapist to perform the exercises), may result in a higher-than-average dropout rate. Alternatively, those signing up may be more motivated to change their present situation and/or have an interest in digital technology and, consequently, show better results. It can also be argued that patients that enroll in the program are currently experiencing an exacerbation in pain and, therefore, are more motivated than the average OA patient. However, this did not seem to be the case for all patients in this study. Patients that enrolled in Joint Academy had a baseline mean NRS pain of 5.1 (SD 2.1) meaning that the pain level of at least some patients was relatively low. Overall, we believe it can be argued that patients in the study cohort are well suited to be the future target group for digital OA management. Despite these limitations, the results encouraged us to further develop Joint Academy. In the current version, we have included extended assessments at inclusion and also during the course of the program. Furthermore, we have included a functional test, comorbidities, and additional demographics to enable an improved user definition in order to further individualize the program. With respect to the enormous OA population, we believe that Joint Academy has the potential to attract people who are more motivated by digital health than by visits to a primary care practice.

In conclusion, we demonstrate that Joint Academy, a Web-based platform for OA therapy, has the potential to successfully deliver individualized Web-based treatment to many patients with OA who presently lack access to treatment.

## Abbreviations

BMI: body mass index
ICHOM: International Consortium for Health Care Measurement
NRS: Numeric Rating Scale
OA: osteoarthritis
SOASP: Supported Osteoarthritis Self-management Program
TJR: total joint replacement
VAS: visual analog scale
